# Bio-Inspired Fault Diagnosis for Aircraft Fuel Pumps Using a Cloud-Edge System

**DOI:** 10.3390/biomimetics8080601

**Published:** 2023-12-13

**Authors:** Yang Miao, Yantang Li, Jun Pan, Zhen Liu, Lei Liu, Zeng Wang, Zijing Wang

**Affiliations:** 1 Faculty of Materials and Manufacturing, Beijing University of Technology, Beijing 100124, China; 2 Beijing Key Laboratory of Advanced Manufacturing Technology, Beijing University of Technology, Beijing 100124, China; 3 AVIC Nanjing Electromechanical Hydraulic Engineering Center, Nanjing 211102, China; 4 Land Space Technology Huzhou Co., Ltd., Huzhou 313099, China; 5 China Aerospace Science and Technology Corporation, Beijing 100076, China; 6 Beijing Institute of Radio Measurement, Beijing 100143, China

**Keywords:** aircraft fuel pump, health monitoring, cloud-edge collaboration, 3/5 strategy, fault diagnosis

## Abstract

The fuel pump serves as the central component of the aircraft fuel system, necessitating real-time data acquisition for monitoring purposes. As the number of sensors increases, there is a substantial rise in data volume, leading to a simultaneous increase in computational processing for traditional Prognostics and Health Management methods while computational efficiency decreases. In response to this challenge, a novel health monitoring approach for aircraft fuel pumps is proposed based on the collaborative utilization of cloud-edge resources. This approach enables efficient cooperation among the sensor side, edge side, and cloud side to achieve timely fault warnings and accurate fault classification for fuel pumps. Within this method, anomaly judgment tasks are allocated to the edge side, and an anomaly judgment method that integrates the 3σ threshold and “3/5 strategy” is devised. Additionally, a fault diagnosis algorithm, founded on a convolutional auto-encoder, is formulated in the cloud to discern various fault types and severities. Comparative results demonstrate that, in contrast to long short-term memory networks, convolutional neural networks, extreme learning machines, and support vector machines, the proposed method yields improvements in accuracy of 4.35%, 6.40%, 17.65%, and 19.35%, respectively. Consequently, it is evident that the proposed method exhibits notable efficacy in the condition monitoring of aircraft fuel pumps.

## 1. Introduction

The fuel pump assumes a crucial role within the fuel system of aircraft, serving as a fundamental component responsible for delivering the requisite pressure and fuel flow to the engine. Monitoring the operational integrity of the fuel pump is of paramount importance, as it significantly contributes to upholding the stability of the fuel system. The existing methods like simulation analysis [1,2], fault tree analysis [3], and signal pro-cessing [4,5] can be used to analyze the fault mechanism of the fuel pump and its rela-tionship.

The emergence of Prognostics and Health Management (PHM) has been inextricably linked with the advent of information technology [6,7,8,9]. It represents a novel approach to health state management that draws on the latest research findings of contemporary information and artificial intelligence technologies, with particular emphasis on equipment health state monitoring, prediction, and management. PHM has served as a catalyst for a pivotal transition in equipment diagnosis, from “post-maintenance” and “scheduled maintenance” to “situational maintenance”. Currently, PHM has found diverse applications in the diagnosis of different devices [10,11,12,13,14]. Especially in the field of aircraft, the application of PHM is much more than common. Che et al. [15] present a PHM model that combines multiple deep learning algorithms for condition assessment, fault classification, sensor prediction, and remaining useful life (RUL) estimation of aircraft systems. Hsu et al. [16] proposed a case study on the implementation of a health assessment and prediction workflow for RUL based on the PHM framework of currently in-service aircraft, which could significantly benefit fleet operators and aircraft maintenance. Yan et al. [17] combined the existing maintenance management experience and the output result of PHM to make more reliable verification for the maintenance of aircraft air-condition systems.

The fundamental approach of PHM involves the utilization of sensors dispersed throughout the equipment to gather operational data. This data are then subjected to analysis through relevant algorithms, aiming to extract valuable information that reflects the equipment’s status, identifies fault modes, and evaluates the remaining lifespan of its components. While most of the health monitoring systems mentioned in the aforementioned literature rely on centralized processing of sensor data, the complexity of equipment structures and the increase in key measurement points have resulted in exponential growth in operational data volume. Consequently, traditional PHM systems experience significant delays in centralized data processing.

The introduction of edge computing [18] effectively addresses this issue. Cloud-edge collaboration has emerged as a crucial research area within the domain of device health management [19,20]. It entails the use of edge computing to share a portion of the computational load with the cloud. This approach not only mitigates the lengthy network transmission delays associated with uploading computational tasks to the cloud but also enables real-time feedback of device abnormalities to technicians. Erbao [21] et al. have successfully addressed the issues of low efficiency and high energy consumption inherent in traditional online monitoring methods for high-voltage equipment in substations by constructing an integrated cloud-edge system. Qiu et al. [22] have analyzed the relationship between various forms of edge computing and industrial edge computing systems while exploring key technologies within the industrial edge computing framework. Li et al. [23] have designed an intelligent fault diagnosis system based on cloud-edge collaboration, employing a fault diagnosis algorithm that combines short-duration memory networks and multi-scale convolutional networks. This system has been applied to machine tool detection, meeting accuracy requirements while reducing diagnosis time.

However, in the aforementioned methods, the monitoring targets primarily consist of individual types of information, such as electrical signals and vibration signals, whereas the monitoring of fuel pumps often involves multiple signal types, like vibration signals and pressure signals. Researchers have also proposed advanced fault diagnosis methods for fuel pumps. For instance, Verhulst et al. [24] developed a fault detection and diagnosis (FDD) scheme to isolate damaged injectors in internal combustion engines. Additionally, Pecho et al. [25] explored the application of a novel technique, Recurrence Quantification Analysis (RQA), for monitoring non-linear transient accelerometer data, specifically for use in auxiliary aircraft hardware health monitoring. A method of intermittent fault diagnosis based on support vector machines (SVM) is presented by Jiang et al. [26] to quickly and effectively diagnose intermittent faults in aircraft fuel pumps. Miao et al. [27,28] addressed the challenges of limited fault data and low diagnostic accuracy of certain new aircraft fuel pumps by deploying the transfer learning method. This approach enabled feature extraction from similar fault data and established an auxiliary dataset to bolster fault diagnosis accuracy, despite the dearth of available data.

This study proposes a cloud-edge collaborative monitoring method specifically for aircraft fuel pumps, incorporating multiple types of information to ensure real-time monitoring effectiveness and accuracy. The main work is as follows:

1. Divide the different tasks on the sensor side, the edge side, and the cloud side to achieve collaborative monitoring;
2. The anomaly judgment method and fault diagnosis algorithm are designed for edge-side state detection and cloud fault classification;
3. The feasibility and effectiveness of the proposed method are verified by fuel pump simulation experiments.

## 2. Fault Diagnosis Framework for Fuel Pumps Based on Cloud Edge Collaboration

### 2.1. General Framework

**Figure 1:**
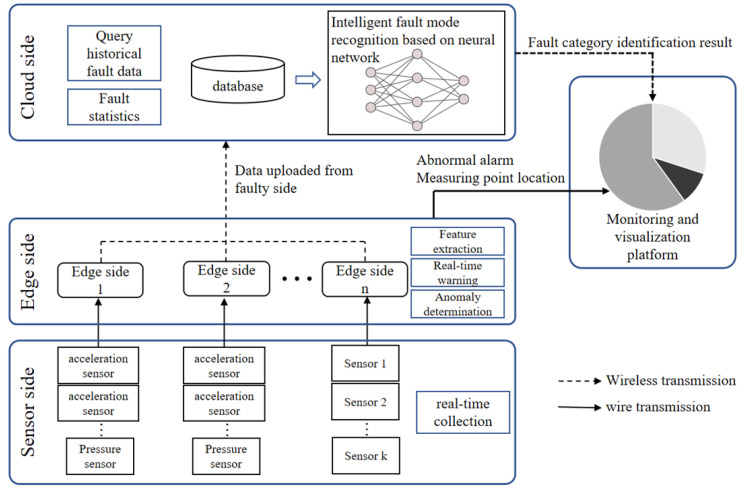
Fuel pump fault diagnosis framework based on cloud-edge collaboration.

A fault diagnosis method for aircraft fuel pumps based on cloud-edge collaboration was established. The methodology comprises three principal components: the cloud side, the edge side, and the sensor side. Each component assumes distinct responsibilities and engages in collaborative relationships, as elucidated in Figure 1.

1. Sensor side: At critical measurement locations of the fuel pump, multiple sensors (e.g., vibration, pressure, etc.) are strategically positioned to capture real-time operating data. These sensors facilitate the collection of comprehensive information regarding the fuel pump’s performance. The acquired real-time data are subsequently transmitted via wired connections to the edge side for further processing and analysis.
2. Edge side: Upon receiving the uploaded data from each sensor, the location information of the corresponding measurement points is recorded, and the characteristic values are extracted simultaneously. Subsequently, the threshold method is employed to identify anomalies within the dataset. Segments of data that meet the abnormal condition are wirelessly transmitted to the cloud for further analysis. In the ground and airborne visual interfaces, both the abnormal alarm information and the respective positions of the measurement points are presented, providing a comprehensive overview of the detected anomalies.
3. Cloud side: The fault segment information transmitted by each edge is stored in the historical database, which facilitates subsequent fault history tracing. An intelligent classification model is employed to identify the types of fault data detected by the system. Additionally, uploading more fault data are used to update and train the model parameters, thus enhancing its migration ability. Ultimately, the classification and diagnosis results are presented through the ground and airborne visual interfaces.

### 2.2. Data Acquisition on Sensor Side

**Figure 2:**
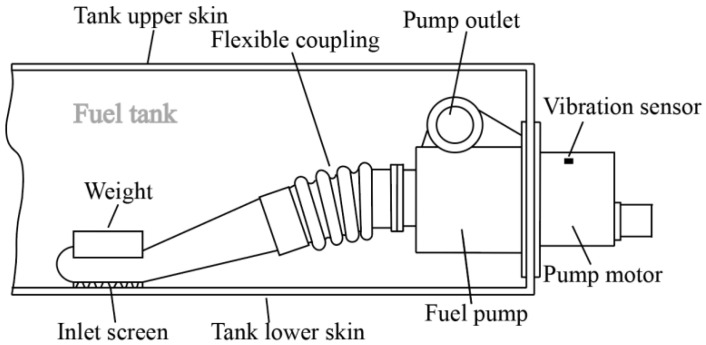
Schematic diagram of the experimental setup.

**Figure 3:**
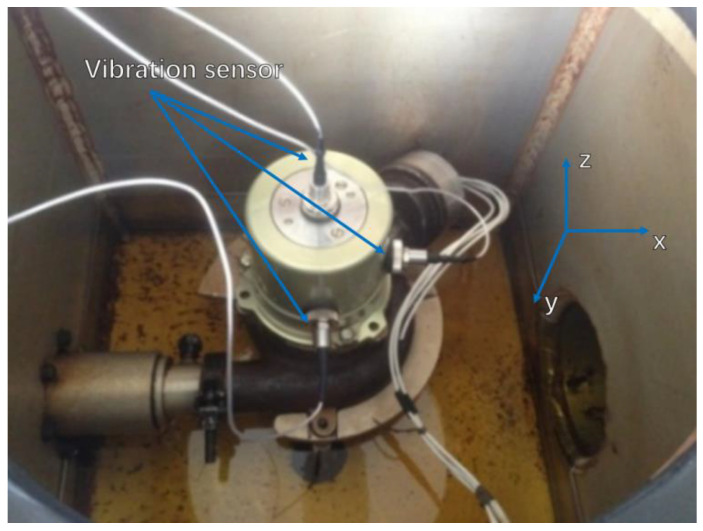
Real picture of the experimental setup.

In order to develop a specialized cloud-edge collaborative fault diagnosis system for fuel pumps, this study establishes a ground-based experimental setup that closely emulates the operational characteristics of an authentic aircraft fuel system. The schematic representation of this apparatus is presented in Figure 2. Given the imperative need to capture vibration and pressure data, a pressure sensor is installed at the oil outlet. Owing to the constrained space within the fuel pump, the direct installation of a vibration sensor poses a challenge. As a result, the vibration sensor is positioned on the motor connected to the fuel pump. The arrangement of the vibration sensor is depicted in Figure 3.

### 2.3. Anomaly Detection on Edge Side

#### 2.3.1. Detection Index

The sensor side is responsible for collecting a range of signals, which are subsequently transmitted to the edge side for preliminary data analysis. On the edge side, an initial analysis is conducted, utilizing the vibration and pressure signals obtained from the sensor side as the primary basis for establishing distinct detection indices.

The direct analysis of the original vibration signals presents certain drawbacks that primarily stem from the large volume of signals collected within a given time frame. These concerns include an increased computational burden at the edge side due to the sheer quantity of original signals, as well as delayed upload speed and subsequent high latency in real-time cloud monitoring when transmitting fault data for pattern recognition at the cloud level, owing to the increased data volume. In order to address these concerns, time-domain indicators are adopted as the fundamental criteria for anomaly detection, allowing for the upload of sample data to the cloud. A comprehensive analysis evaluating the sensitivity and stability of time-domain features is presented in Table 1.

Based on the information presented in the aforementioned table, it can be observed that the kurtosis factor and margin factor exhibit high sensitivity and are particularly responsive to the occurrence of faults. On the other hand, the root-mean-square demonstrates good stability and effectively assesses changes in vibration signals. The comprehensive performance of the peak and peak-to-peak indices is superior. Taking into account the overall computational complexity, five key indices, namely peak, peak-to-peak, root mean square, kurtosis factor, and margin factor, are selected as the primary characteristics for determining abnormal conditions in fuel pump assessment.

The peak and peak-to-peak indices capture the maximum amplitude of the vibration wave pattern, making them well-suited for detecting surface stripping faults. These types of faults generate signals with significant values within a short time period, which can be mathematically described by Equations (1)–(3):

(1) $$X_{\max}=\max(x_{i})$$

(2) $$X_{\min}=\min(x_{i})$$

(3) $$X_{peak}=\frac{1}{2}(X_{\max}-X_{\min})$$

where *x_i_* is the signal for a period of time, and *X_peak_* is the peak value.

The root-mean-square reflects the energy distribution of the signal in the time domain, expressed by Equation (4):

(4) $$X_{rms}=\sqrt{\frac{1}{n}\sum_{i=1}^{n}x_{i}^{2}}$$

where *X_rms_* is the root-mean-square value.

The kurtosis factor is sensitive to shock characteristics in the signal, as expressed by Equations (5) and (6):

(5) $$X_{k}=\frac{\sum_{i=1}^{n}x_{i}^{4}}{n}$$

(6) $$X_{kf}=\frac{X_{k}}{X_{rms}^{4}}$$

where *X_k_* is kurtosis and *X_kf_* is the kurtosis factor.

The margin factor is more suitable for early impact faults, and its expression is as in Equations (7) and (8):

(7) $$X_{r}={\left(\frac{\sum_{i=1}^{n}{\left|x_{i}\right|}^{\frac{1}{2}}}{n}\right)}^{2}$$

(8) $$X_{cl}=\frac{X_{peak}}{X_{r}}$$

where *X_r_* is the margin factor and *X_cl_* is the root amplitude.

In the context of anomaly detection, pressure signals exhibit a higher degree of discernibility compared to vibration signals. During normal operation of the fuel pump, pressure information tends to fluctuate around the expected normal value. However, in the presence of anomalies, such as instances of low or high pressure, the pressure information deviates significantly, reaching excessively high levels or persisting at abnormal values for a prolonged duration. Consequently, the analysis of pressure signals primarily revolves around assessing the amplitude and duration of such deviations.

It has been observed that pressure signals exhibit a higher degree of discernibility compared to vibration signals. In normal operational scenarios, pressure information tends to fluctuate around the expected normal value. However, in the presence of anomalous events, such as instances of low or high pressure, the pressure information deviates significantly from the norm, reaching excessively high levels or persisting at abnormal values for prolonged periods. Therefore, the analysis of pressure signals primarily revolves around assessing the amplitude and duration of such deviations.

#### 2.3.2. Threshold Detection

For the abnormal judgment of a vibration signal, the 3σ threshold judgment method is used, which can be expressed as Equation (9):

(9) $$f(x)=\frac{1}{\sigma \sqrt{2\pi }}\exp[-\frac{1}{2}{\left(\frac{x-\mu }{\sigma }\right)}^{2}],\;-\infty <x<\infty$$

where *σ* is the population standard deviation and *μ* is the population mean.

**Figure 4:**
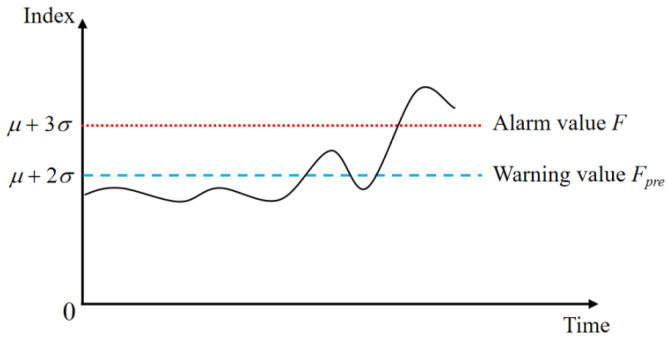
Vibration time domain characteristic threshold judgment.

By examining the properties of the probability density curve associated with a normal distribution, it becomes evident that the likelihood of a random variable following a normal distribution outside the interval (*μ* − 3*σ*, *μ* + 3*σ*) is merely 0.26%. This probability, commonly known as the 3σ rule, characterizes the occurrence of values within this range in a normal distribution. Figure 4 presents a schematic representation depicting the threshold judgment of vibration signals.

Upon reaching the predetermined warning threshold value *F_pre_*, it signifies the emergence of a fault trend at the specific measurement point. Consequently, timely attention or maintenance is necessary during subsequent operations. Conversely, when the indicator surpasses the alarm threshold value *F*, it indicates that the measurement point is unlikely to meet the requirements for stable operation. In such cases, prompt remedial measures should be taken to rectify the fault.

**Figure 5:**
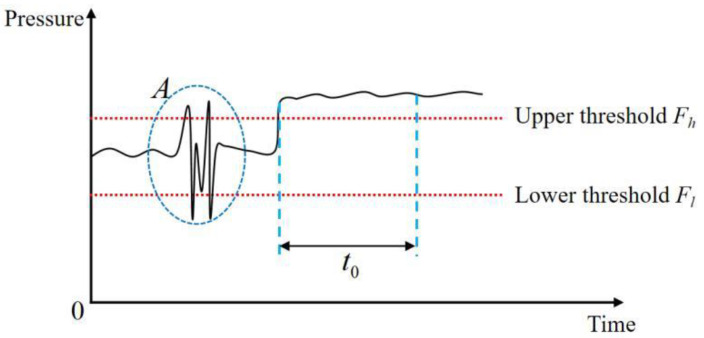
Pressure threshold judgment.

The pressure detection is shown in Figure 5.

During the normal operation of fuel pumps, internal pressure is anticipated to remain in close proximity to a predetermined fixed value. Therefore, the establishment of upper and lower thresholds assumes paramount importance for evaluating whether the pressure deviates excessively, indicating abnormal functioning. Nonetheless, the assessment process must factor in the presence of pressure pulsation phenomena [29]. Such pulsations can cause pressure excursions beyond the upper threshold or lower than the lower threshold, as demonstrated by location A in Figure 5. To prevent false-positive occurrences resulting from pressure anomalies induced solely by pressure pulsations, an additional condition is introduced. Specifically, an alarm trigger is activated when the pressure surpasses the threshold and remains beyond a specified duration, thus ensuring more accurate identification of abnormal pressure instances.

#### 2.3.3. 3/5 Strategy

The identification of abnormal signals plays a crucial role in facilitating the early detection of anomalies, thereby triggering warning and alarm systems. However, when dealing with multiple measuring points within the fuel pump, each equipped with different indicators, relying on a single instance of abnormality per indicator to trigger alarms can lead to considerable confusion for monitoring personnel and result in increased extraneous workload.

Considering these factors, the employed abnormal alarm strategy in this investigation adopts the following approach: treating the measuring point as the fundamental unit, alarms are repetitively triggered when multiple indicators within the vibration signals exhibit abnormal behavior. In the context of pressure signals, alarms are activated only when abnormalities persist for an extended period of time.

To account for potential variations among the five indicators within the random vibration signal, where it is possible for not all indicators to simultaneously exceed the alarm threshold, a methodology is devised to ensure comprehensive detection of anomalies. Specifically, when four out of the five indicators concurrently surpass the alarm threshold, it is recorded as an occurrence of an anomaly. It is important to note that individual indicators, such as the margin factor, may occasionally exceed the threshold value even under normal operating conditions. To prevent such instances from hindering accurate anomaly identification, a “3/5 strategy” is formulated. This strategy involves the establishment of five storage windows, collectively forming a detection stage, in which the detection results from each instance are stored within their respective window. Subsequently, if three out of the five consecutive detection results indicate anomalies, an alarm is triggered, and relevant feedback is transmitted to the designated terminal.

Regarding the pressure information, its assessment involves determining whether the pressure amplitude exceeds the upper or lower thresholds and maintaining this condition for a duration of time denoted as *t*. If the duration t exceeds a predefined threshold value *t*_0_, the pressure information at the measuring point is classified as abnormal.

### 2.4. Cloud Fault Pattern Recognition Algorithm Based on Convolutional Auto-Encoder

While real-time anomaly detection and alarm notifications on a human-computer interaction platform can serve as initial steps towards effective data monitoring, they prove inadequate for the development of a comprehensive health monitoring system. The scarcity and value of fault data pertaining to aircraft fuel pumps emphasize the need for an intelligent fault classification model within a cloud-based environment. This approach offers multiple benefits by maximizing the utilization of uploaded data and providing more comprehensive fault information, including the category and severity of abnormalities. Such detailed information supports subsequent maintenance tasks with enhanced precision.

**Figure 6:**
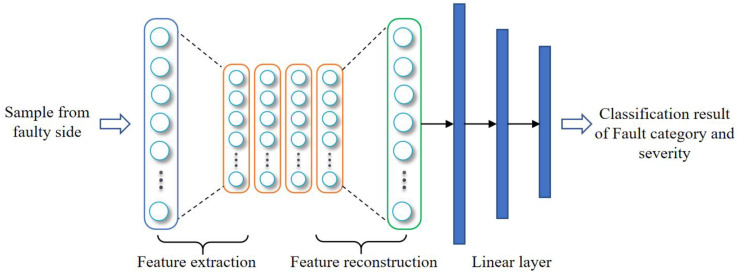
Structure diagram of a cloud fault diagnosis algorithm based on CAE.

The convolutional auto-encoder (CAE) is a commonly employed technique for unsupervised feature extraction and the preservation of feature invariance [30]. Expanding upon this notion, the present study introduces a fault classifier constructed using a cloud-based CAE. The architectural framework of the proposed model is illustrated in Figure 6.

The fault data uploaded from the edge devices originates from the fuel pump during normal operational conditions, hence incorporating some degree of noise within the data samples. CAE is an effective way to mitigate the disruptive impact of noise on the sample characteristics. It is structured with an input layer, multiple hidden layers, and an output layer, enabling effective utilization of unsupervised learning techniques to extract efficient representations of the input samples. Its operational procedure primarily consists of two stages: feature extraction and feature reconstruction. During feature extraction, the input samples are transformed into high-dimensional feature information through operations such as convolution and pooling. Subsequently, in the process of feature reconstruction, the high-dimensional encoded information is deconvolved, thereby converting it into low-dimensional information. Importantly, it should be emphasized that the reconstructed sample information exhibits more pronounced features compared to the input data, rendering it more amenable to distinguishing fault categories. The specific process of the fault diagnosis algorithm in this paper is as follows:

The fault data uploaded from the edge devices originates from the fuel pump during normal operational conditions, which introduces some degree of noise into the data samples. To mitigate the disruptive effects of this noise on the sample characteristics, CAE is an effective approach that is structured with an input layer, multiple hidden layers, and an output layer, which enable unsupervised learning techniques to extract efficient representations of the input samples. Its operational procedure primarily consists of two stages: feature extraction and feature reconstruction. During feature extraction, the input samples are transformed into high-dimensional feature information through operations such as convolution and pooling. Subsequently, in the process of feature reconstruction, the high-dimensional encoded information is deconvolved, thereby converting it into low-dimensional information. It is worth noting that the reconstructed sample information exhibits more pronounced features compared to the input data, rendering it more suitable for distinguishing fault categories. The specific process of the fault diagnosis algorithm in this paper proceeds as follows:

1. Feature extraction process

A convolution operation is performed to initialize *k* convolution kernels, each of which is paired with a paranoid. After the convolution of the input fault sample *x*, *k* features are generated. The ReLU function is used as the activation function, expressed by Equation (10):

(10) $$h^{k}=\sigma (x*W^{k}+b^{k})$$

where *W* is the convolution kernel and *b* is paranoid.

Following the generation of features using multiple convolution operations, these features undergo pooling while retaining their position relation matrix. This facilitates subsequent de-pooling operations. After multiple convolution and pooling operations, the sample information is transformed into high-dimensional feature information. This high-dimensional information is then subjected to de-convolution and de-pooling operations, resulting in its reconstruction into a new sample.

2. Feature reconstruction process

In order to de-pool the high-dimensional feature matrix that is generated during the process of feature extraction, it is necessary to restore the data to its original size and position by utilizing the retained position relation matrix. This is achieved by performing a convolution operation on the transposition of each feature and its corresponding convolution kernel, followed by the summation of the results and the addition of the bias term c. The ReLU activation function is still employed, and its mathematical expression is given by Equation (11):

(11) $$y=\sigma (\Sigma_{k}h^{k}*W^{-k}+c)$$

where *c* is paranoid.

The minimum mean square error (MSE) function is used as a loss function to update network weights. MSE can be expressed as:

(12) $$E(\theta )=\frac{1}{2n}\sum_{i=1}^{n}{\left(x_{i}-y_{i}\right)}^{2}$$

where *θ* is network weight.

3. Classifier

After CAE, the input sample is updated into a new sample with more obvious features, and then three linear layers L1, L2, and L3 are connected. The linear layer can be expressed as Equation (13):

(13) $$y_{i}=w_{ij}*x_{i-1}+b_{ij}$$

where *x_i_*_−1_ is the input data, *y_i_* is the output data, and *w_ij_* and *b_ij_* are the weights and biases, respectively.

Finally, the diagnosis result will be output by connecting to the softmax classifier.

## 3. Experiment and Result Analysis

### 3.1. Experimental Setup

**Figure 7:**
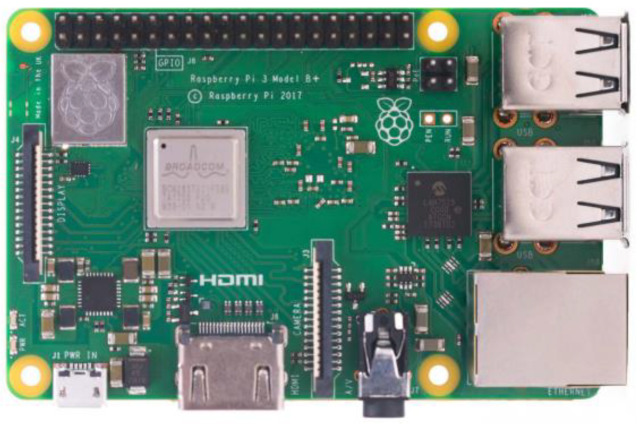
Raspberry Pi 3 Model B+.

The experimental platform employed in this study is established within the framework of the Key Laboratory of Aeronautical System Integration and Aviation Technology at the Nanjing Mechanical Hydraulic Engineering Technology Research and Development Center. The platform encompasses various components, including a centrifugal gasoline pump, oil supply tank, oil storage tank, vibration sensor, pressure sensor, and other related apparatus. Its primary function entails facilitating the transportation of fuel from the storage tank to the supply cylinder. At the edge level, the Raspberry Pi 3B+ (depicted in Figure 7) is utilized for data anomaly detection. Moreover, the cloud-based fault diagnosis algorithm is developed using the PyTorch deep learning platform. Upon processing the collected data, a total of 800 samples are obtained, which are subsequently partitioned into 600 training samples and 200 test samples. Notably, each sample consists of 2048 data points, thereby providing a comprehensive dataset for subsequent analysis and evaluation.

The experimental platform utilized in this research is situated within the framework of the Key Laboratory of Aeronautical System Integration and Aviation Technology at the Nanjing Mechanical Hydraulic Engineering Technology Research and Development Center. This platform comprises a range of components, including a centrifugal gasoline pump, oil supply tank, oil storage tank, vibration sensor, pressure sensor, and other relevant equipment. Its primary purpose is to facilitate the transfer of fuel from the storage tank to the supply cylinder. At the edge side, the Raspberry Pi 3B+ (depicted in Figure 7) is employed for data anomaly detection. Furthermore, the fault diagnosis algorithm deployed in the cloud is developed using the PyTorch deep learning platform. After processing the collected data, a total of 800 samples are acquired, which are subsequently divided into 600 training samples and 200 test samples. Notably, each sample comprises 2048 data points, thereby furnishing a comprehensive dataset for subsequent analysis and evaluation.

### 3.2. Result of Edge Side Detection

**Figure 8:**
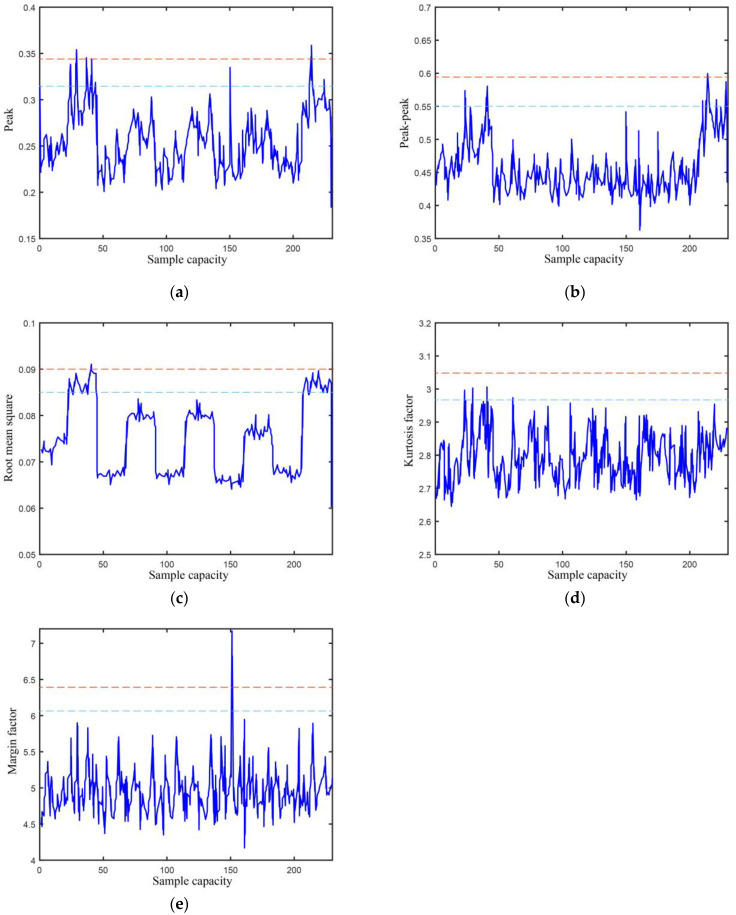
Characteristic change in the time domain. The red line indicates the alarm threshold, while the blue line indicates the warning threshold. (**a**) Peak; (**b**) Peak-to-peak; (**c**) Root mean square; (**d**) Kurtosis factor; (**e**) Margin factor.

Concurrently with the acquisition of vibration data, the time-domain index of the signal is computed at regular intervals of 0.1 s to ensure real-time monitoring of each index. Specifically, Figure 8 illustrates the temporal evolution of several parameters, including peak, peak-to-peak, root mean square, kurtosis factor, margin factor warning threshold, and alarm threshold, over a designated time period. As observed in the figure, the peak, peak-to-peak, root mean square, and margin factor all exceed their respective alarm thresholds within this period, while the kurtosis factor does not. Consequently, this instance is classified as an anomaly and recorded within the storage window for further analysis.

### 3.3. Performance Comparison of Different Fault Diagnosis Algorithms for Fuel Pumps

To validate the efficacy of the CAE-based fuel pump fault diagnosis algorithm, this study conducts a comparative analysis with other techniques, including support vector machines (SVM), extreme learning machines (ELM), convolutional neural networks (CNN), and long short-term memory networks (LSTM). The evaluation criteria employed for this analysis comprise accuracy, precision, recall, and F1 score, which are defined by (14)–(17):

(14) $$Accuracy=\frac{TP+TN}{TP+TN+FP+FN}$$

(15) $$Precision=\frac{TP}{TP+FP}$$

(16) $$Recall=\frac{TP}{TP+FN}$$

(17) $$F1-score=\frac{2TP}{2TP+FP+FN}$$

where TP represents the number of positive samples correctly classified as positive samples and FP represents the number of negative samples misclassified as positive samples; TN represents the number of negative samples correctly classified as negative samples; FN represents the number of positive samples that are misclassified as negative.

**Figure 9:**
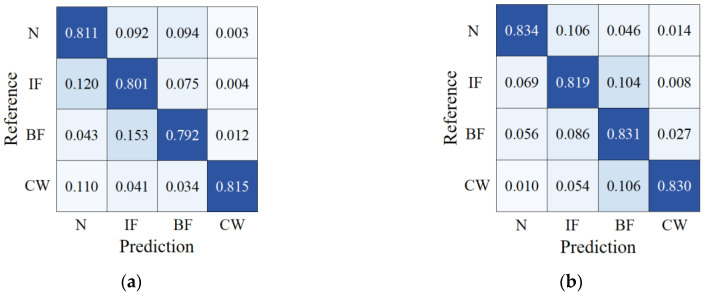

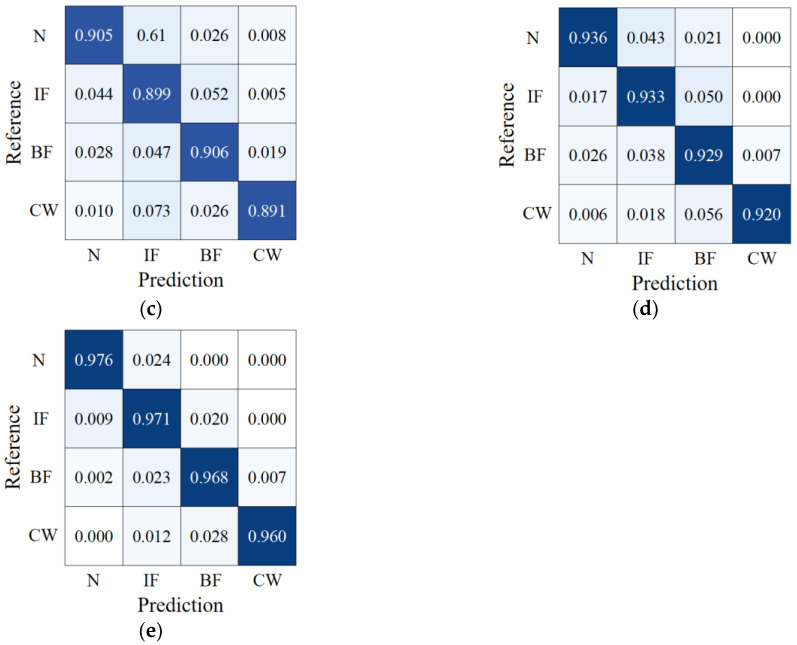
Confusion matrix: (**a**) SVM; (**b**) ELM; (**c**) CNN; (**d**) LSTM; (**e**) CAE.

A total of five experiments were conducted for each of the fault diagnosis algorithms, and the resulting average values for each index were computed. The confusion matrixes are presented in Figure 9, and the comparative outcomes are presented in Table 2. As demonstrated in the table, the CAE-based fuel pump fault diagnosis algorithm proposed in this study demonstrates superior performance on four indices. In terms of accuracy rate, for instance, compared to LSTM, CNN, ELM, and SVM, the proposed approach achieved improvements of 4.35%, 6.40%, 17.65%, and 19.35%, respectively. The noteworthy results can be mainly attributed to the potent feature extraction and reconstruction capabilities of the CAE. In the presence of the challenging operational conditions of the fuel pump, direct signal feature extraction may result in the fusion of noise information with useful data, leading to inaccurate fault diagnosis outcomes. However, the proposed methodology integrates the CAE to reconstruct the extracted deep features into new samples, which contain more informative details and prominent features and are less susceptible to noise interference. During the classification process, the weight of noise information is mitigated, leading to improved diagnostic accuracy.

### 3.4. Analysis of the Influence of Sample Number on Cloud Diagnosis Algorithm

**Figure 10:**
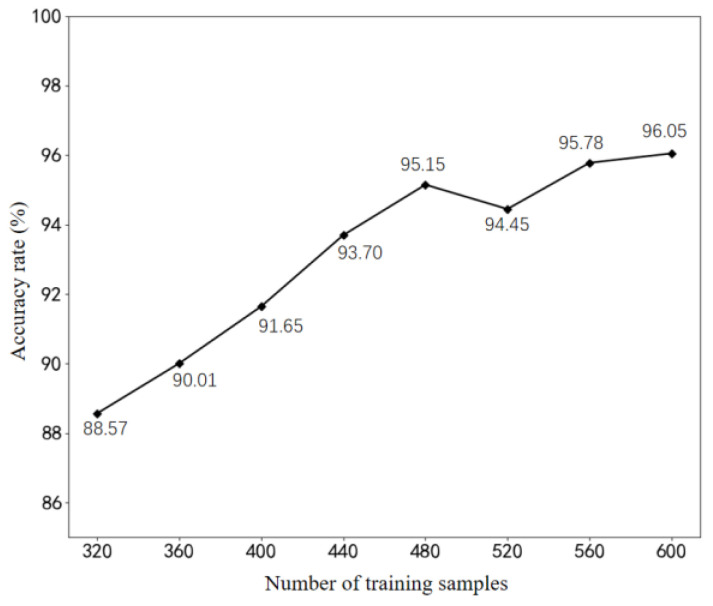
Relationship between the number of training samples and test accuracy.

Following anomaly detection at the edge, the data segments associated with faults are transmitted to the cloud for fault classification and severity diagnosis. Subsequently, the diagnosis outcomes, along with the corresponding fault data and labels, are stored in the historical database. This database serves as the primary data source for training and enhancing model performance. As the training data volume increases, the diagnostic model acquires a greater knowledge base from the samples, thereby impacting its diagnostic accuracy. This study investigates the influence of varying numbers of training samples on the model’s detection accuracy. A series of experiments were conducted, testing additional sets of 40 samples each, ranging from 320 to 600 samples. Five experiments were performed for each test, and the results were averaged. Figure 10 illustrates the diagnostic accuracy results achieved by training the model with different sample sizes. The analysis reveals that the model’s detection accuracy rate improves with an increasing number of training samples within the range of 320 to 480. However, beyond 480 training samples, the growth rate of accuracy diminishes, fluctuating between 95% and 96%.

As the detection time extends, the quantity of fault data uploaded to the cloud not only increases but also encompasses the same faults occurring under different working conditions. Incorporating these fault samples for updating the cloud model can enhance the model’s capability to detect faults across diverse working conditions and improve its overall generalization performance.

## 4. Conclusions

In this study, a cloud-edge collaborative intelligent monitoring scheme is proposed for the purpose of monitoring aircraft fuel pumps. The scheme leverages edge and cloud computing to perform anomaly detection and fault diagnosis tasks, respectively. This approach effectively reduces the computational burden associated with performing real-time monitoring under all-weather conditions. To maintain high alert accuracy while minimizing false positives, the 3σ threshold is combined with a “3/5 strategy” at the edge. To classify and identify fault features, the cloud employs the CAE machine learning method. Experimental testing conducted on a fuel pump experiment platform demonstrates that CAE outperforms other methods such as SVM, ELM, CNN, and LSTM in terms of accuracy and stability. The proposed cloud-edge intelligent monitoring scheme for aircraft fuel pumps successfully addresses issues such as large data volumes, low detection efficiency, and difficulty in fault identification often encountered with traditional fault diagnosis methods. As such, this scheme provides a promising foundation for establishing a robust aircraft fuel pump health monitoring system.

## Figures and Tables

**Table 1:** Sensitivity and stability of time domain features.

Time Domain Index	Sensitivity	Stability
Peak	Preferably	Common
Peak-to-peak	Common	Common
Mean	Worse	Preferably
Root-mean-square	Preferably	Preferably
Kurtosis factor	Well	Common
Waveform factor	Bad	Well
Margin factor	Well	Common
Skewness	Common	Worse
Pulse factor	Common	Common
Peak factor	Common	Common

**Table 2:** Performance comparison of fuel pump fault diagnosis algorithms.

Model	Accuracy	Precision	Recall	F1-Score
SVM	76.70 ± 1.67	80.12 ± 1.39	76.70 ± 1.28	76.40 ± 1.20
ELM	78.40 ± 0.97	82.35 ± 0.71	78.40 ± 1.32	77.63 ± 1.25
CNN	89.65 ± 0.77	89.99 ± 0.96	89.65 ± 1.02	89.75 ± 0.86
LSTM	91.70 ± 1.04	92.69 ± 0.87	91.70 ± 2.06	91.45 ± 1.65
CAE (proposed)	96.05 ± 0.62	96.46 ± 0.63	96.05 ± 0.99	96.03 ± 0.33

## Data Availability

The data that supports the findings of this study are available from the corresponding author upon reasonable request.
